# [(1,3-Benzothia­zol-2-yl)amino­carbon­yl]meth­yl piperidine-1-carbodithio­ate monohydrate

**DOI:** 10.1107/S1600536811018460

**Published:** 2011-05-25

**Authors:** Xu-Jia Lu, Hong-Bin Zhao, Liang Chen, De-Liang Yang, Bang-Ying Wang

**Affiliations:** a Department of Organic Chemistry, the College of Chemistry, Xiangtan University, Hunan 411105, People’s Republic of China; b Environmental Engineering, Dongguan University of Technology, Guangdong 523808, People’s Republic of China

## Abstract

In the title compound, C_15_H_17_N_3_OS_3_·H_2_O, the piperidine ring has a chair conformation. The crystal structure is stabilized by weak inter­molecular N—H⋯O, O—H⋯N and O—H⋯O hydrogen-bonding inter­actions.

## Related literature

For the biological activity of substituted *N*-benzothia­zol-2-yl-amides, see: Patel & Shaikh (2010 ▶); Hou *et al.* (2006 ▶). For related structures, see: Wang *et al.* (2008 ▶).
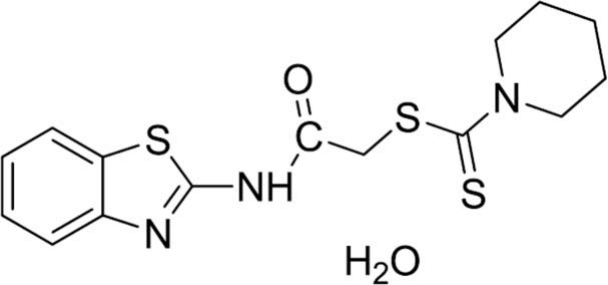


## Experimental

### 

#### Crystal data

C_15_H_17_N_3_OS_3_·H_2_O
*M* *_r_* = 369.39
Monoclinic, 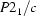

*a* = 10.6326 (3) Å
*b* = 12.0735 (3) Å
*c* = 14.7824 (4) Å
β = 113.133 (2)°
*V* = 1745.08 (8) Å^3^
*Z* = 4
Mo *K*α radiation
μ = 0.44 mm^−1^
*T* = 296 K
0.20 × 0.20 × 0.20 mm

#### Data collection

Bruker SMART APEXII CCD area-detector diffractometer
Absorption correction: multi-scan (*SADABS*; Sheldrick, 2004 ▶) *T*_min_ = 0.918, *T*_max_ = 0.918
15147 measured reflections
3992 independent reflections
3497 reflections with *I* > 2σ(*I*)
*R*_int_ = 0.020

#### Refinement

*R*[*F* ^2^ > 2σ(*F* ^2^)] = 0.034
*wR*(*F* ^2^) = 0.095
*S* = 1.11
3992 reflections
202 parameters
H-atom parameters constrained
Δρ_max_ = 0.21 e Å^−3^
Δρ_min_ = −0.30 e Å^−3^

### 

Data collection: *APEX2* (Bruker, 2004 ▶); cell refinement: *SAINT* (Bruker, 2004 ▶); data reduction: *SAINT*; program(s) used to solve structure: *SHELXS97* (Sheldrick, 2008 ▶); program(s) used to refine structure: *SHELXL97* (Sheldrick, 2008 ▶); molecular graphics: *SHELXTL* (Sheldrick, 2008 ▶); software used to prepare material for publication: *SHELXTL*.

## Supplementary Material

Crystal structure: contains datablocks global, I. DOI: 10.1107/S1600536811018460/fj2417sup1.cif

Structure factors: contains datablocks I. DOI: 10.1107/S1600536811018460/fj2417Isup2.hkl

Supplementary material file. DOI: 10.1107/S1600536811018460/fj2417Isup3.cml

Additional supplementary materials:  crystallographic information; 3D view; checkCIF report

## Figures and Tables

**Table 1:** Hydrogen-bond geometry (Å, °)

*D*—H⋯*A*	*D*—H	H⋯*A*	*D*⋯*A*	*D*—H⋯*A*
N2—H3⋯O2^i^	0.84	1.91	2.745 (2)	170
O2—H2′*A*⋯N3^ii^	0.92	2.04	2.920 (2)	160
O2—H2′*B*⋯O1^iii^	0.91	1.92	2.821 (2)	169

Symmetry codes: (i) 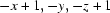; (ii) 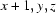; (iii) 
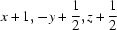
.

**Fig. 1.**
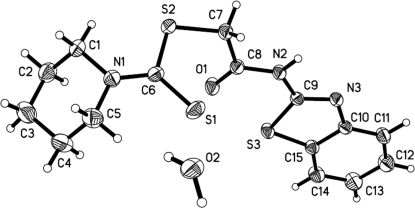
A view of (I), with the atom-labeling scheme and 30% probability displacement ellipsoids.

**Fig. 2.**
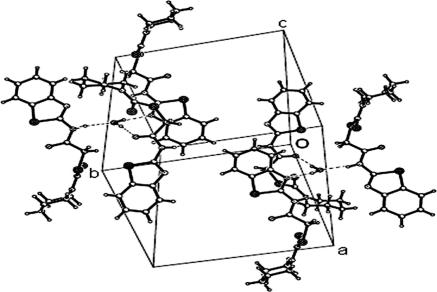
Packing of the title compound. Dashed lines indicate hydrogen bonds.

## supplementary crystallographic information

### Comment

Substituted *N*-benzothiazol-2-yl-amides are an important class of heterocyclic compounds that exhibit a wide range of biological properties such as antimicrobial activity (Patel & Shaikh, 2010), antivirus infections (Hou *et al.*, 2006). In this paper, the structure of 1-(dithiopiperidyl)-*N*-benzothiazole-2-yl-acetamide is reported.

The title compound (I) (Fig. 1) crystallizes in the centrosymmetric space group P21/c. One 1-(dithiopiperidyl)-*N*-benzothiazole-2-yl-acetamide molecule and a solvent water molecule in the asymmetric unit. The piperidine ring has a chair conformation; Crystal packing is stablized by N—H···O, O—H···N and O—H···O hydrogen bonds (Figs. 2 and Table 1).

### Experimental

Single crystals were recrystallization from an ethanol solution at room temperature.

### Refinement

H atoms were placed in calculated positions (C—H=0.93–0.97 Å, N—H=0.85 Å,*O*—H=0.91–0.92 Å) and refined in riding mode, with Uĩso~(H) = xU~eq~(C,*N*), where *x* =1.5 (O,*N*) and 1.2 for all other H atoms.

### Crystal data

**Table:**

C_15_H_17_N_3_OS_3_·H_2_O	*F*(000) = 776
*M**_r_* = 369.39	*D*_x_ = 1.406 Mg m^−^^3^
Monoclinic, *P*2_1_/*c*	Mo *K*α radiation, λ = 0.71073 Å
Hall symbol: -P 2ybc	Cell parameters from 8358 reflections
*a* = 10.6326 (3) Å	θ = 3.0–27.5°
*b* = 12.0735 (3) Å	µ = 0.44 mm^−^^1^
*c* = 14.7824 (4) Å	*T* = 296 K
β = 113.133 (2)°	Block, colorless
*V* = 1745.08 (8) Å^3^	0.20 × 0.20 × 0.20 mm
*Z* = 4	

### Data collection

**Table:**

Bruker APEXII CCD area-detector diffractometer	3992 independent reflections
Radiation source: fine-focus sealed tube	3497 reflections with *I* > 2σ(*I*)
graphite	*R*_int_ = 0.020
φ and ω scans	θ_max_ = 27.5°, θ_min_ = 3.0°
Absorption correction: multi-scan (*SADABS*; Sheldrick, 2004)	*h* = −12→13
*T*_min_ = 0.918, *T*_max_ = 0.918	*k* = −14→15
15147 measured reflections	*l* = −19→19

### Refinement

**Table:**

Refinement on *F*^2^	Primary atom site location: structure-invariant direct methods
Least-squares matrix: full	Secondary atom site location: difference Fourier map
*R*[*F*^2^ > 2σ(*F*^2^)] = 0.034	Hydrogen site location: inferred from neighbouring sites
*wR*(*F*^2^) = 0.095	H-atom parameters constrained
*S* = 1.11	*w* = 1/[σ^2^(*F*_o_^2^) + (0.0424*P*)^2^ + 0.6208*P*] where *P* = (*F*_o_^2^ + 2*F*_c_^2^)/3
3992 reflections	(Δ/σ)_max_ < 0.001
202 parameters	Δρ_max_ = 0.21 e Å^−^^3^
0 restraints	Δρ_min_ = −0.30 e Å^−^^3^

### Special details

**Table:**

Geometry. All e.s.d.'s (except the e.s.d. in the dihedral angle between two l.s. planes) are estimated using the full covariance matrix. The cell e.s.d.'s are taken into account individually in the estimation of e.s.d.'s in distances, angles and torsion angles; correlations between e.s.d.'s in cell parameters are only used when they are defined by crystal symmetry. An approximate (isotropic) treatment of cell e.s.d.'s is used for estimating e.s.d.'s involving l.s. planes.
Refinement. Refinement of *F*^2^ against ALL reflections. The weighted *R*-factor *wR* and goodness of fit *S* are based on *F*^2^, conventional *R*-factors *R* are based on *F*, with *F* set to zero for negative *F*^2^. The threshold expression of *F*^2^ > σ(*F*^2^) is used only for calculating *R*-factors(gt) *etc*. and is not relevant to the choice of reflections for refinement. *R*-factors based on *F*^2^ are statistically about twice as large as those based on *F*, and *R*- factors based on ALL data will be even larger.

### Fractional atomic coordinates and isotropic or equivalent isotropic displacement parameters (Å^2^)

**Table:**

	*x*	*y*	*z*	*U*_iso_*/*U*_eq_	
S3	−0.11155 (5)	0.39341 (3)	0.39288 (3)	0.04084 (12)	
S2	0.14706 (5)	0.19374 (3)	0.16270 (3)	0.04125 (12)	
S1	0.34529 (6)	0.24704 (5)	0.36726 (3)	0.05848 (15)	
O1	0.00460 (15)	0.32824 (10)	0.26734 (10)	0.0505 (3)	
N3	−0.14307 (15)	0.20161 (10)	0.46198 (10)	0.0387 (3)	
N1	0.37483 (17)	0.30273 (14)	0.20291 (11)	0.0504 (4)	
N2	−0.02961 (10)	0.18869 (7)	0.35648 (7)	0.0398 (3)	
H3	−0.0250	0.1209	0.3715	0.060*	
C15	−0.19188 (10)	0.38955 (7)	0.47513 (7)	0.0390 (3)	
C14	−0.24377 (10)	0.47707 (7)	0.51145 (7)	0.0524 (4)	
H14	−0.2375	0.5497	0.4927	0.063*	
C13	−0.3046 (2)	0.45284 (17)	0.57595 (18)	0.0621 (5)	
H13	−0.3404	0.5099	0.6008	0.075*	
C12	−0.3132 (2)	0.34427 (18)	0.60443 (17)	0.0607 (5)	
H12	−0.3539	0.3300	0.6486	0.073*	
C11	−0.2626 (2)	0.25758 (15)	0.56845 (15)	0.0506 (4)	
H11	−0.2696	0.1852	0.5874	0.061*	
C10	−0.20087 (17)	0.28022 (13)	0.50311 (12)	0.0381 (3)	
C9	−0.09440 (16)	0.24949 (12)	0.40459 (11)	0.0344 (3)	
C8	0.01508 (18)	0.23052 (13)	0.28907 (12)	0.0389 (3)	
C7	0.0712 (2)	0.14189 (14)	0.24244 (13)	0.0450 (4)	
H7A	−0.0025	0.0920	0.2054	0.054*	
H7B	0.1392	0.0992	0.2943	0.054*	
C6	0.30123 (18)	0.25403 (13)	0.24634 (12)	0.0401 (3)	
C1	0.3334 (2)	0.31304 (17)	0.09552 (13)	0.0524 (5)	
H1A	0.2466	0.2758	0.0618	0.063*	
H1B	0.4010	0.2776	0.0763	0.063*	
C2	0.3200 (2)	0.43266 (18)	0.06582 (15)	0.0607 (5)	
H2A	0.2454	0.4658	0.0785	0.073*	
H2B	0.2983	0.4380	−0.0042	0.073*	
C3	0.4502 (3)	0.4956 (2)	0.1215 (2)	0.0794 (7)	
H3A	0.4357	0.5738	0.1057	0.095*	
H3B	0.5218	0.4697	0.1016	0.095*	
C4	0.4948 (3)	0.4796 (2)	0.23209 (18)	0.0701 (6)	
H4A	0.5832	0.5144	0.2661	0.084*	
H4B	0.4295	0.5156	0.2534	0.084*	
C5	0.5044 (2)	0.3595 (2)	0.25880 (17)	0.0632 (5)	
H5A	0.5774	0.3253	0.2449	0.076*	
H5B	0.5261	0.3520	0.3287	0.076*	
O2	0.99835 (17)	0.03537 (10)	0.61071 (10)	0.0591 (4)	
H2'A	0.9501	0.0735	0.5538	0.089*	
H2'B	1.0111	0.0822	0.6617	0.089*	

### Atomic displacement parameters (Å^2^)

**Table:**

	*U*^11^	*U*^22^	*U*^33^	*U*^12^	*U*^13^	*U*^23^
S3	0.0527 (3)	0.02621 (18)	0.0499 (2)	0.00354 (15)	0.0270 (2)	0.00531 (15)
S2	0.0520 (3)	0.0395 (2)	0.0354 (2)	−0.00204 (17)	0.02063 (18)	−0.00248 (15)
S1	0.0800 (4)	0.0582 (3)	0.0310 (2)	0.0053 (2)	0.0152 (2)	0.00416 (19)
O1	0.0772 (9)	0.0309 (6)	0.0565 (8)	0.0079 (6)	0.0403 (7)	0.0086 (5)
N3	0.0505 (8)	0.0279 (6)	0.0419 (7)	−0.0021 (5)	0.0225 (6)	0.0002 (5)
N1	0.0531 (9)	0.0581 (9)	0.0363 (7)	−0.0110 (7)	0.0135 (7)	0.0000 (7)
N2	0.0581 (9)	0.0250 (6)	0.0431 (7)	0.0033 (6)	0.0273 (7)	0.0030 (5)
C15	0.0415 (8)	0.0336 (8)	0.0442 (8)	0.0010 (6)	0.0192 (7)	0.0000 (6)
C14	0.0612 (11)	0.0352 (8)	0.0684 (12)	0.0057 (8)	0.0336 (10)	−0.0015 (8)
C13	0.0706 (13)	0.0503 (11)	0.0828 (15)	0.0061 (9)	0.0490 (12)	−0.0094 (10)
C12	0.0674 (13)	0.0575 (12)	0.0762 (14)	−0.0045 (10)	0.0487 (12)	−0.0074 (10)
C11	0.0603 (11)	0.0418 (9)	0.0611 (11)	−0.0072 (8)	0.0362 (10)	−0.0022 (8)
C10	0.0407 (8)	0.0330 (7)	0.0426 (8)	−0.0021 (6)	0.0185 (7)	−0.0021 (6)
C9	0.0405 (8)	0.0261 (7)	0.0359 (7)	0.0006 (6)	0.0144 (6)	0.0010 (5)
C8	0.0495 (9)	0.0318 (8)	0.0386 (8)	0.0030 (6)	0.0209 (7)	0.0021 (6)
C7	0.0632 (11)	0.0318 (8)	0.0496 (9)	0.0011 (7)	0.0325 (9)	0.0010 (7)
C6	0.0515 (9)	0.0328 (7)	0.0343 (8)	0.0058 (7)	0.0151 (7)	0.0012 (6)
C1	0.0619 (12)	0.0603 (11)	0.0391 (9)	−0.0108 (9)	0.0243 (8)	−0.0040 (8)
C2	0.0705 (14)	0.0633 (13)	0.0498 (11)	0.0025 (10)	0.0252 (10)	0.0052 (9)
C3	0.0941 (19)	0.0622 (14)	0.0802 (16)	−0.0202 (13)	0.0324 (14)	0.0070 (12)
C4	0.0686 (14)	0.0641 (13)	0.0749 (15)	−0.0181 (11)	0.0252 (12)	−0.0150 (12)
C5	0.0502 (11)	0.0731 (14)	0.0565 (12)	−0.0111 (10)	0.0103 (9)	−0.0025 (11)
O2	0.1018 (11)	0.0269 (6)	0.0487 (7)	0.0058 (6)	0.0297 (7)	0.0001 (5)

### Geometric parameters (Å, °)

**Table:**

S3—C15	1.7395 (10)	C11—C10	1.392 (2)
S3—C9	1.7486 (15)	C11—H11	0.9300
S2—C6	1.7757 (18)	C8—C7	1.517 (2)
S2—C7	1.7828 (17)	C7—H7A	0.9700
S1—C6	1.6624 (16)	C7—H7B	0.9700
O1—C8	1.2163 (19)	C1—C2	1.500 (3)
N3—C9	1.291 (2)	C1—H1A	0.9700
N3—C10	1.394 (2)	C1—H1B	0.9700
N1—C6	1.329 (2)	C2—C3	1.508 (3)
N1—C5	1.468 (3)	C2—H2A	0.9700
N1—C1	1.477 (2)	C2—H2B	0.9700
N2—C8	1.3586 (18)	C3—C4	1.525 (3)
N2—C9	1.3799 (17)	C3—H3A	0.9700
N2—H3	0.8445	C3—H3B	0.9700
C15—C14	1.3936	C4—C5	1.496 (3)
C15—C10	1.3978 (18)	C4—H4A	0.9700
C14—C13	1.377 (2)	C4—H4B	0.9700
C14—H14	0.9300	C5—H5A	0.9700
C13—C12	1.391 (3)	C5—H5B	0.9700
C13—H13	0.9300	O2—H2'A	0.9186
C12—C11	1.376 (3)	O2—H2'B	0.9083
C12—H12	0.9300		
			
C15—S3—C9	87.99 (6)	S2—C7—H7B	108.6
C6—S2—C7	102.54 (8)	H7A—C7—H7B	107.6
C9—N3—C10	109.94 (13)	N1—C6—S1	124.85 (14)
C6—N1—C5	122.35 (16)	N1—C6—S2	113.75 (12)
C6—N1—C1	124.96 (15)	S1—C6—S2	121.40 (10)
C5—N1—C1	112.56 (16)	N1—C1—C2	110.48 (16)
C8—N2—C9	124.72 (11)	N1—C1—H1A	109.6
C8—N2—H3	123.5	C2—C1—H1A	109.6
C9—N2—H3	111.7	N1—C1—H1B	109.6
C14—C15—C10	121.23 (8)	C2—C1—H1B	109.6
C14—C15—S3	128.8	H1A—C1—H1B	108.1
C10—C15—S3	109.98 (9)	C1—C2—C3	111.35 (19)
C13—C14—C15	118.06 (9)	C1—C2—H2A	109.4
C13—C14—H14	121.0	C3—C2—H2A	109.4
C15—C14—H14	121.0	C1—C2—H2B	109.4
C14—C13—C12	120.96 (16)	C3—C2—H2B	109.4
C14—C13—H13	119.5	H2A—C2—H2B	108.0
C12—C13—H13	119.5	C2—C3—C4	110.69 (19)
C11—C12—C13	121.18 (18)	C2—C3—H3A	109.5
C11—C12—H12	119.4	C4—C3—H3A	109.5
C13—C12—H12	119.4	C2—C3—H3B	109.5
C12—C11—C10	118.79 (17)	C4—C3—H3B	109.5
C12—C11—H11	120.6	H3A—C3—H3B	108.1
C10—C11—H11	120.6	C5—C4—C3	111.4 (2)
C11—C10—N3	125.33 (15)	C5—C4—H4A	109.3
C11—C10—C15	119.77 (14)	C3—C4—H4A	109.3
N3—C10—C15	114.89 (13)	C5—C4—H4B	109.3
N3—C9—N2	120.70 (13)	C3—C4—H4B	109.3
N3—C9—S3	117.19 (12)	H4A—C4—H4B	108.0
N2—C9—S3	122.09 (10)	N1—C5—C4	110.63 (18)
O1—C8—N2	122.14 (14)	N1—C5—H5A	109.5
O1—C8—C7	125.17 (15)	C4—C5—H5A	109.5
N2—C8—C7	112.63 (13)	N1—C5—H5B	109.5
C8—C7—S2	114.45 (12)	C4—C5—H5B	109.5
C8—C7—H7A	108.6	H5A—C5—H5B	108.1
S2—C7—H7A	108.6	H2'A—O2—H2'B	107.2
C8—C7—H7B	108.6		
			
C9—S3—C15—C14	179.51 (6)	C15—S3—C9—N2	−178.26 (14)
C9—S3—C15—C10	−0.94 (11)	C9—N2—C8—O1	2.2 (3)
C10—C15—C14—C13	−0.05 (14)	C9—N2—C8—C7	−175.06 (14)
S3—C15—C14—C13	179.45 (15)	O1—C8—C7—S2	8.9 (3)
C15—C14—C13—C12	0.4 (3)	N2—C8—C7—S2	−173.96 (12)
C14—C13—C12—C11	−0.7 (4)	C6—S2—C7—C8	71.97 (15)
C13—C12—C11—C10	0.6 (3)	C5—N1—C6—S1	−1.7 (3)
C12—C11—C10—N3	178.85 (19)	C1—N1—C6—S1	−177.38 (15)
C12—C11—C10—C15	−0.3 (3)	C5—N1—C6—S2	178.72 (16)
C9—N3—C10—C11	−179.96 (17)	C1—N1—C6—S2	3.1 (2)
C9—N3—C10—C15	−0.8 (2)	C7—S2—C6—N1	−177.71 (13)
C14—C15—C10—C11	0.00 (19)	C7—S2—C6—S1	2.73 (12)
S3—C15—C10—C11	−179.59 (14)	C6—N1—C1—C2	118.0 (2)
C14—C15—C10—N3	−179.20 (9)	C5—N1—C1—C2	−58.0 (2)
S3—C15—C10—N3	1.21 (17)	N1—C1—C2—C3	55.5 (2)
C10—N3—C9—N2	178.87 (14)	C1—C2—C3—C4	−53.5 (3)
C10—N3—C9—S3	0.03 (19)	C2—C3—C4—C5	53.3 (3)
C8—N2—C9—N3	174.61 (15)	C6—N1—C5—C4	−118.4 (2)
C8—N2—C9—S3	−6.6 (2)	C1—N1—C5—C4	57.7 (2)
C15—S3—C9—N3	0.55 (13)	C3—C4—C5—N1	−54.9 (3)

### Hydrogen-bond geometry (Å, °)

**Table:**

*D*—H···*A*	*D*—H	H···*A*	*D*···*A*	*D*—H···*A*
N2—H3···O2^i^	0.84	1.91	2.745 (2)	170
O2—H2'A···N3^ii^	0.92	2.04	2.920 (2)	160
O2—H2'B···O1^iii^	0.91	1.92	2.821 (2)	169

Symmetry codes: (i) −*x*+1, −*y*, −*z*+1; (ii) *x*+1, *y*, *z*; (iii) *x*+1, −*y*+1/2, *z*+1/2.
